# Effects of 8 Weeks of High-Intensity Interval Training and Spirulina Supplementation on Immunoglobin Levels, Cardio-Respiratory Fitness, and Body Composition of Overweight and Obese Women

**DOI:** 10.3390/biology11020196

**Published:** 2022-01-26

**Authors:** Hadi Nobari, Elham Aminifar, Jalil Reisi, Reyhaneh Vahabidelshad, Katsuhiko Suzuki, Stella Lucia Volpe, Jorge Pérez-Gómez

**Affiliations:** 1 Department of Exercise Physiology, Faculty of Educational Sciences and Psychology, University of Mohaghegh Ardabili, Ardabil 56199-11367, Iran; 2 Department of Physiology, School of Sport Sciences, University of Extremadura, 10003 Cáceres, Spain; 3 Department of Exercise Physiology, Faculty of Sport Sciences, University of Isfahan, Isfahan 81746-7344, Iran; elham_eynii@yahoo.com; 4 Faculty of Motor Sciences, Université Libre de Bruxelles, 1070 Brussels, Belgium; reyhanevahabi@yahoo.com; 5 Faculty of Sport Sciences, Waseda University, Saitama 359-1192, Japan; katsu.suzu@waseda.jp; 6 Department of Human Nutrition, Foods and Exercise, Virginia Polytechnic Institute and State University, Blacksburg, VA 24061, USA; stellalv@vt.edu; 7 HEME Research Group, Faculty of Sport Sciences, University of Extremadura, 10003 Cáceres, Spain; jorgepg100@gmail.com

**Keywords:** antioxidant, body fat, IgA, immunomodulation, nutritional supplement, obesity, physical activity

## Abstract

**Simple Summary:**

Overweight and obese, like other forms of malnutrition, have been shown to affect immune function through changing immunoglobin or cardio-respiratory fitness levels and cell-mediated immune responses. Although calorie restriction and exercise are the most common therapies for obesity or overweight, it is unclear what kind of supplementation these people should take or how much exercise they should perform. Hence, in this study, we examined the effect of 8 weeks of high-intensity interval training (HIIT) with spirulina supplementation on the humoral immunity, cardio-respiratory fitness, and body composition of overweight and obese women. The results demonstrated that spirulina supplementation with HIIT not only decreased fat free mass but also boosted immunoglobin-A, which plays an important role in the immune system.

**Abstract:**

Our study examined the effect of 8 weeks of high-intensity interval training (HIIT) and spirulina supplementation on the humoral immunity, cardio-respiratory fitness, and body composition of overweight and obese women. Thirty sedentary women (height: 161.7 ± 2.8 cm, body mass: 75.8 ± 8.4 kg, body mass index [BMI]: 28.8 ± 2.5 kg/m^2^, age: 25.1 ± 6.7 years) were divided into three groups: placebo with HIIT group, spirulina group (SG), or combined group (CG). Exercise groups performed HIIT for 8 weeks, with three sessions per week and four to seven repetitions in each session of 30 s running and 30 s walking; the intensity was established at 90% of the maximum heart rate. Supplementation groups received 6 g of spirulina powder per day. Fasting blood samples were collected before and after 8 weeks to determine the concentrations of immunoglobulins (IgA and IgG). There was a significant group-by-time interaction for fat free mass (FFM; *p* = 0.001, f = 8.52, ηp^2^ = 0.39) and IgA (*p* = 0.036, f = 3.86, ηp^2^ = 0.22). The post hoc analysis revealed that CG reduced FFM significantly (*p* = 0.012, *g* = −0.55) after training. CG and SG showed significantly greater IgA concentrations after 8 weeks (*p* = 0.02, *g* = 0.70 and *p* = 0.001, *g* = 0.34, respectively). We conclude that spirulina supplementation with HIIT affects the body composition (lower FFM) but also boosts IgA, which plays an important role in the immune system.

## 1. Introduction

Overweight and obesity refer to the abnormal or excessive accumulation of fat that may lead to an increased risk of chronic disease. The World Health Organization (WHO) defines a body mass index (BMI) ≥ 25 kg/m^2^ as overweight and a BMI ≥ 30 kg/m^2^ as obesity [1,2]. Research has shown that, with each unit of increase in BMI, the risk of cardiovascular disease increases by 8%. However, for each unit of a metabolic equivalent task increase in physical activity, the risk of cardiovascular disease decreases. Excess body fat is a condition associated with an impaired immune system and greater susceptibility to developing an infectious disease [3,4,5].

*Spirulina maxima* is used as a nutritional supplement because of its phytochemical content (phenolic compounds, carotenoids, and tocopherols) and essential nutrients (proteins, *n*-3 and *n*-6 fatty acids) [6,7]. It has been suggested that spirulina might help to increase lean body mass because of its high protein content, particularly of the branched-chain amino acids, leucine, valine, and isoleucine. As a result of this, athletes have used spirulina to improve the body composition and physical performance [8,9]. Among the species of spirulina that are safe for consumption are *Spirulina maxima*, arthrospira fusiformis, and platensis, and the latter is the most commonly used and studied in the scientific literature [10]. As previously stated, spirulina has a high protein content (50% to 70% of its dry weight) [11], all of the essential amino acids, most of the vitamins and minerals, and it confers numerous health benefits, such as antioxidant, immunomodulatory, anti-inflammatory, and antiviral activities [7,12]. Many athletes have consumed spirulina for these health benefits, and it was suggested that the Chinese and Cuban Olympic teams consumed spirulina daily for many years to improve their athletic performance [13]. Hernández-Lepe et al. gave 4.5 g of spirulina per day to their participants for six weeks [14]. 

High-intensity interval training (HIIT), which is alternating between periods of high intensity and recovery, has become a popular training method due to its time efficiency. HIIT is effective for improving fasting blood glucose concentrations and reducing blood pressure in overweight or obese populations [15,16]. Recent studies have clearly shown that intense intermittent exercise is better for reducing fat than endurance exercise [17,18,19]. In addition, HIIT has been shown to reduce blood pressure in individuals who are overweight or obese [20]. HIIT has been shown to have benefits in young and older individuals on body weight, the regulation of physiological parameters such as blood pressure, improvement in aerobic capacity as measured by maximum oxygen consumption (VO_2max_), and reductions in glucose and triglyceride concentrations and fat free mass, with an increased lower limb muscle power [21]. HIIT is also effective for improving fasting glucose concentrations and reducing blood pressure in individuals who are overweight or obese [22]. In a study of 20 healthy untrained overweight/obese males, the following 12 weeks of HIIT reported that the BMI and fat mass percentage were significantly decreased [23]. HIIT also increase cardiopulmonary fitness. The best indicator for assessing cardiorespiratory fitness is the measurement of VO₂max [24]. Gillen et al. [25] showed that short-term low-volume HIIT is a time-efficient strategy to improve the body composition and muscle oxidative capacity in women who are overweight or obese. In addition, Andreato et al. [26] reported that HIIT can be used as a secondary method for the treatment of obesity in adults.

The immune system contains complex mechanisms that are of particular importance in the body’s defense against pathogenic microorganisms, bacteria, parasites, and viruses. The immune system is divided into two arms: innate immunity (natural or non-special) and adaptive immunity (acquired or special), where the acquired immunity is divided into humoral and cellular parts. Cellular immunity includes T cells (e.g., CD₈, CD₄, and CD₃) and B cells (e.g., CD₂₂, CD₂₀, and CD₁₉). Humoral immunity includes immunoglobulins (e.g., IgM, IgA, IgD, IgE, and IgG) [27]. IgA is the major immunoglobulin in mucous secretions, such as saliva and tears, and it is thought to provide a front line of defense against pathogens and antigens present on mucosal surfaces, such as the airways. IgA is able to inhibit the binding of viruses and bacteria to the mucosal epithelium and viral replication [28]. Controversial results have been observed regarding the impact of HIIT on the immune system. The majority of the studies have shown that HIIT suppressed the immune system, whereas others have reported that this training did not affect the immune system [27,29,30]. In a few cases, it has been reported that HIIT improved immune function [31,32]. IgG is vital for the proper functioning of the body’s defense system, staying healthy, and fighting pathogens [33]. Owen et al. [34] showed that high-intensity soccer training might cause a significant decrease in s-IgA values post-exercise compared to low-intensity training. Lee et al. [35] indicated that there was a reduced trend of IgA in male adults after 12 weeks of judo training alone, or combined with resistance training or with interval training. The initial laboratory examination of humoral immunity consists of measuring the levels of various immunoglobulin (IgG and IgA) in serum [36]. The mean values for IgG were from 720 to 1038 mg/100 mL in the females that we can compare with our study [37]. In our study, overweight and obese women were recruited because there are few studies about HIIT and spirulina supplementation on the humoral immune system, cardio-respiratory fitness, and body composition in this population. Therefore, we hypothesized that 8 weeks of HIIT and spirulina supplementation could affect the humoral immune system, VO_2max_, and body composition in overweight and obese women.

## 2. Materials and Methods

### 2.1. Participants

**Figure 1:**
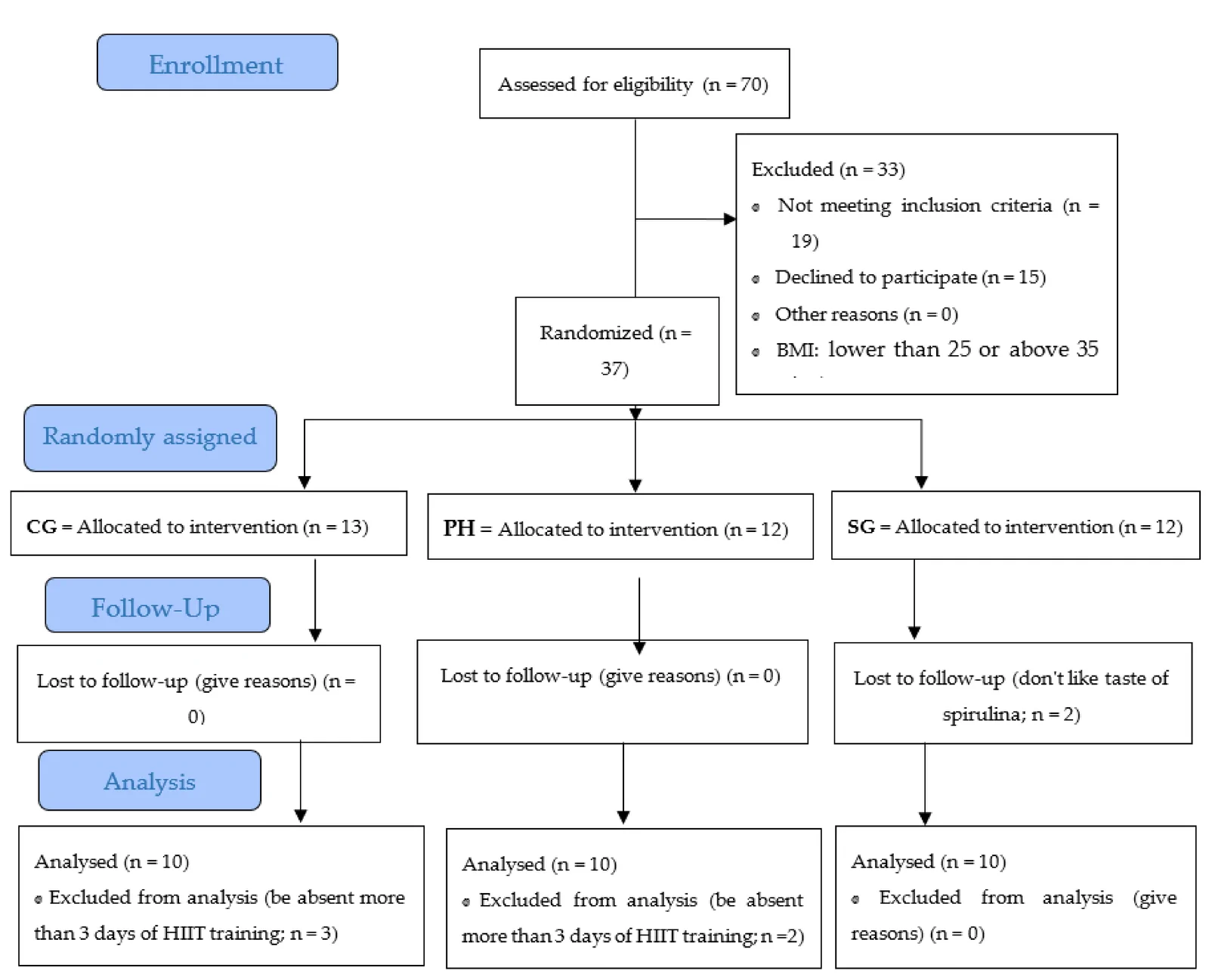
Flow diagram of how to enter, experimental course, and analysis of participants. Abbreviation; PH = placebo and high-intensity interval training (HIIT); CG = combined group (spirulina and HIIT); SG = spirulina group; BMI = body mass index.

Thirty women (mean ± standard deviation); 25.1 ± 6.7 years of age; height: 161.7 ± 2.8 cm, body mass: 75.8 ± 8.4 kg; with a BMI between 25 to 35 kg/m^2^, were divided into three groups: placebo with HIIT (PH, *n* = 10), spirulina group (SG, *n* = 10), and combined group (CG, *n* = 10). PH performed HIIT for 8 weeks, 3 sessions per week, and received a placebo per day. SG received 6 g of spirulina powder per day and did not participate in any regular training. CG performed the same HIIT and received 6 g of spirulina powder per day [38]. Exclusion and enrollment criteria for all groups were: (1) participants who were physically active; (2) participants who were on a weight loss diet; (3) participants with medical conditions for physical activity; (4) participants with a BMI lower than 25 or above 35 kg/m^2^; (5) they should not have any specific illness or diet; (6) those who missed more than three practice sessions were excluded from the study (Figure 1). All participants signed and accepted the informed consent according to the recommendations of the Helsinki Declaration for Human Research. Our study is a part of the master’s thesis of the University of Isfahan registered with the code IR.UI.REC.1397.145.

Within 24 h following the treatment, subjects were advised not to ingest alcohol, caffeine, theine, hot liquids, or smoke. In addition, subjects were advised not to ingest medications, performance-enhancing capsules, or other supplements during the study [39]. We instructed the participants to maintain their usual dietary intake and not to lower their energy intake, and also asked them to maintain their usual physical activity during the study [40]. Based on the recommendation, participants maintained their usual dietary intake and physical activity levels.

### 2.2. Sample Size

Using the statistical method investigated and G-Power software (University of Dusseldorf, Dusseldorf, Germany), we calculated the design’s power and sample size. This included the following: a priori and F tests are used to calculate the achieved power; ANOVA: repeated measurements, within-interaction analysis; err prob for α = 0.05; minimum effect size = 0.35; number of groups = 3; number of measures = 2; and err prob for 1-β = 0.80. With real or actual power, there is an 82.2% chance of successfully rejecting the null hypothesis of no difference in variables in the study with 24 participants.

### 2.3. Experimental Approach to the Problem

This research was a quasi-experimental, double-blinded design with baseline and post-intervention measurements. Before starting the research, age, height, and BMI were assessed (Table 1), and BF% was measured by the thickness of the subcutaneous fat layer using skinfold analysis. In the next stage, participants performed a shuttle run test to assess aerobic fitness according to guidelines [41] of VO_2max_ measurement. Participants received oral and written information about supplement. Blood samples were collected at two different time points: before and after the supplementation and training periods. The samples were transferred to the laboratory immediately after each collection and centrifuged there.

### 2.4. Measurement of Fat Free Mass

Fat free mass (FFM) was calculated with the following formula for women, with weight (W) in kilograms and height (H) in centimeters: FFM = (0.29569 × W) + (0.41813 × H) − 43.2933 [42,43]. To measure body fat percentage, seven subcutaneous fat thicknesses were used by the Jackson and Pollock method [44,45,46]. Seven points were included: triceps, chest, subscapular, suprailiac, axilla, abdominal, and thigh [47]. Data were collected by Lafayette Instrument Company (Lafayette, IN, USA) with an accuracy of 0.1 mm. All measurements were performed by one person on the right side of the body. The person who took the skinfold measurements had taken several skinfold measurements over many years [48,49,50]. The technical measurement error was considered according to previous studies [51,52].

### 2.5. Measurement of Cardio-Respiratory Fitness

VO₂max was measured by 20-m shuttle-run test. The maximum field test consisted of reciprocating runs between two lines 20 m apart at a speed adjusted to a pre-recorded audible alarm [53]. The initial speed set to start the test was 8.5 km/h^−1^, which was increased to 0.5 km/h^−1^ after each minute. Participants were instructed to continue the test to the last step as much as possible. The test ended when the person could not keep up with the running speed, or when the person was unable to reach the 20-m area within each lane three times in a row in accordance with the audible warning. The velocity obtained during the last step that was fully performed was considered as the maximum test velocity, and was calculated as VO₂max by placing it in the following formula [41]: VO₂max (mL·kg^−1^·min^−1^) = 6 (x) − 24.4 X. X is the maximum aerobic speed, which is determined by the running speed at the highest level [54].

### 2.6. Measurement of Blood Samples

To measure IgA and IgG 24 h before and 24 h after the study period, 10 mL blood samples were taken from the left vein of the participants between 9:00 and 11:00 in the morning, blood samples were collected into pipes containing solution of acidic anticoagulant EDTA K2, and, after plasma centrifugation, their plasma was separated, where the resulting plasma was kept at −20 °C. IgA and IgG concentrations were measured by using Hitachi device, laboratory turbidometry, and Pars test kits. The turbidometry method is based on a complex formation resulting from the reaction between immunoglobulins and its specific antiserum. The amount of turbidity generated is directly related to the amount of immunoglobulins. The minimum volume required to measure IgG and IgA by a turbidometric device is 50 μL.

### 2.7. Exercise Protocol

Participants participated in HIIT exercises three times a week for 8 weeks, with an intensity of 90% of maximum heart rate. Exercises started from 24 min in the first session (5 to 10 min of warm-up, 30 s of exercise (running), and 30 s of active rest (walking), with 4 repetitions and 5 to 10 min of cool down), and 27 min in the last session (5 to 10 min of warm-up, 30 s of exercise, and 30 s of active rest, with 7 repetitions and 5 to 10 min of cool down) [55]. The participants of the two training groups performed the training protocol at a distance of 20 m according to Glaister et al. [56]. In the round-robin test practice protocol, participants first ran at maximum speed from the starting point (cone 1) to cone 2 in lane A. After returning in the opposite direction on route B, they ran 20 m towards cone 3 with maximum speed, and finally, after returning, they ran again at maximum speed on route C (cone 1) to complete the distance of 40 m. Participants continued to perform this at maximum speed until the 30-s period of the training protocol ended, and, after a 30-s break, they repeated the training protocol. Exercise progressed by increasing the number of 30-s repetitions from four times in the first and second weeks to five times in the third and fourth weeks, to six times, in the fifth and sixth weeks, and to seven times in the seventh and 8-week of practice, like previous study training protocol [56]. The intensity of training in all stages of the protocol was 90% of the maximum heart rate. The heart rate was measured by a control instructor using a Polar pacemaker made in Finland, and the maximum predictable heart rate was estimated with the formula of Tanaka (208 − 0.7 × age (years)) [57]. All participants participated in the exercises until they were completed. In this study, the participants in the supplement group did not have any regular exercise.

### 2.8. Supplementation

In the present study, spirulina algae powder was prepared from Isfahan Green Agate Company (Isfahan, Iran). The participants in the CG and SG received 6 g per day of water-soluble spirulina powder half an hour before a meal, and the participants in the PH received a green coloring food dissolved in water; this was close to other relevant human studies, as they received 8 g of spirulina per day [58].

### 2.9. Statistical Methods

Data analysis of the present study has been carried out at both descriptive and inferential concentrations. The distribution between the data was also examined by Shapiro–Wilk test. Equality of variance in different groups was also assessed by Levin test. To determine possible group differences pre-training calculated with a one-way analysis of variance (ANOVA), a 2 × 3 ANOVA with repeated measures (time [pre- vs. post-training] × group [CG vs. SG vs. PH]) was used to determine differences between groups, and then we used the suitable Tukey post hoc test when a significant group-by-time interaction was discovered. Hedge’s g effect size with 95% confidence interval was also calculated to determine the magnitude of pairwise comparisons for pre- and post-test, and was defined as trivial (<0.2), small (≥0.02), moderate (≥0.05), and large (≥0.08) [59]. If the results of the one-way ANOVA and effect sizes were similar for each group (i.e., FFM), then the percentage changes were computed and assessed. Significance of statistical analysis was used at the level of *p* < 0.05. All statistical calculations were performed using SPSS (Version 25.0; IBM SPSS Inc., Chicago, IL, USA).

## 3. Results

Table 2 shows the mean and standard deviation of the changes in VO_2max_, IgA, and IgG. At the baseline, there were no differences observed between groups in the above variables (*p* > 0.05). There was no significant main effect of time for IgA (*p* = 0.073, f = 3.48, ηp^2^ = 0.11); however, there was a meaningful group-by-time interaction (*p* = 0.036, f = 3.86, ηp^2^ = 0.22). The post hoc analysis found that IgA (CG, *p* = 0.02, *g* = 0.70 and SG, *p* = 0.001, *g* = 0.34) was significantly greater post-test versus pre-test.

Table 3 shows the mean and standard deviation in the anthropometric and body composition. At the baseline, there were no differences observed between groups in all variables, except the waist-to-hip ratio (f = 4.39, *p <* 0.02). Based on the analysis, there was no significant main effect of time for FFM (*p* = 0.36, f = 0.86, ηp^2^ = 0.03), whereas there was a meaningful group-by-time interaction (*p* = 0.001, f = 8.52, ηp^2^ = 0.39). The post hoc analysis indicated that FFM (kg) (CG, *p* = 0.012, *g* = −0.54) was significantly reduced. In addition, in the SG group, this variable increased but was not significant (*p* > 0.05). However, there were significant main effects of time for the rest of the variables, but there were no significant group-by-time interactions for changes in these variables.

## 4. Discussion

Our purpose was to examine the effect of 8-week HIIT and spirulina supplementation on the humoral immune function, cardio-respiratory fitness, and body composition of overweight and obese women. We found that 8-week HIIT with 6 g of spirulina supplementation per day significantly improved IgA (CG: 212.7 ± 67.8 and SG: 187.5 ± 44.6) compared to the baseline (CG: 173.1 ± 33.7 and SG: 171.1 ± 47.9) and FFM (18.3 ± 1.0) compared to the baseline (18.8 ± 0.8) in CG. Although IgG did not change significantly, the percentage change in the spirulina groups was illustrated as being between 13 to 15%, with a medium-to-high effect size. The study of Jahani et al. [60] that examined the effect of HIIT and probiotic supplementation on immune cells, C-reactive protein, and IgA showed that intense intermittent exercise increases IgA, which is consistent with the results of the present study. Spirulina has a powerful stimulatory effect on the immune system through increasing the phagocytic activity of macro-phages, inducing the accumulation of natural killers cells in tissues, stimulating antibody and cytokine production, and activating and mobilizing T or B cells [61]. Previous studies demonstrated that spirulina diminishes the negative effect of different agents of Ig concentrations [62,63] and leukocyte numbers [64].

The researchers concluded that exercise-induced changes in serum Ig concentrations may be due to the participation of extravascular proteins, increased lymphocytes after exercise, a combination of changes in the plasma volume and extravascular flow, and changes in the subject’s circadian cycle [65]. Factors involved in immunity include sex, age, race, smoking, strenuous or moderate physical activity, alcohol consumption, obesity, pregnancy, hormonal factors, and common microflora in each individual’s digestive tract [66,67]. Therefore, one of the factors involved in the difference between the results of other studies and the findings of the present study can be enumerated in environmental and genetic factors that were beyond the control of the researchers [68]. Saeedy et al. [31] showed that HIIT, along with zinc supplementation, significantly improved IgA. Qieqeshlaq et al. [68] showed that HIIT and probiotic increased IgA significantly. Spirulina has a hypolipidemic activity and decreases the concentrations of liver profiles [69].

Mohebi et al. [70] showed that 8-week high-intensity resistance training decreased IgG concentration significantly in untrained men. The contradiction with the present study may be due to the difference between detailed training and participants. Santoso et al. [71] showed that there was a change between the IgG level of pre- and post-test of breathing arts sports treatment, and that this change increased significantly after the respiratory exercise. Although the IgG concentrations did not alter considerably, the spirulina groups showed a percentage change of 13 to 15% with a medium to large effect size. The phycocyanin in spirulina increases biological activity against infectious diseases by maintaining the function of the mucosal immune system and reducing allergic inflammation by suppressing specific antibodies, and injecting it produces IgA antibodies. Spirulina polysaccharide also activates innate immune cells and increases antibodies [72]. The polysaccharides and phycocyanins in spirulina help to both increase the number of antigens through physical activity and increase the immune system. Therefore, intense intermittent exercise with spirulina supplementation has a greater effect on strengthening the immune system [73,74].

Spirulina supplementation decreased the FFM of overweight and obese women significantly. Hunter et al. showed that, after 4 weeks of resistance training, the amount of FFM decreased significantly [75]. The results of their research is consistent with our results, which is probably due to the similar training duration [76].

Our present study had some limitations. We did not control the participants’ diets. Furthermore, we could have had a longer duration for our study. In addition, by observing large percentage changes in the variable of IgG, we did not see significant changes, which was probably due to individual effects and large changes in some participants. We strongly recommend that individual differences, resting energy expenditure, physical activity levels, and dietary intake be considered in future studies. Additionally, more studies with different ages, as well as with women and men, may help to delineate the effects of HIIT and spirulina supplementation on the immune function, body composition, and exercise performance. Finally, another limitation of the study could be the field training protocol that has been performed. This could be considered in future studies by increasing the control of the training intensity with heart rate and considering the session calibration of the device used.

## 5. Conclusions

In this study, the effect of an 8-week period of HIIT combined with spirulina supplementation on the humoral immune system and body composition of overweight and obese women was investigated. The data in the present study demonstrated the effectiveness of spirulina supplementation and HIIT concurrently in making significant changes in IgA concentrations and FFM. Taking spirulina with HIIT for overweight and obese women may be helpful not only for losing FFM but also for boosting IgA, which plays an important role in the immune system.

## Figures and Tables

**Table 1:** Characteristics of the participants.

Group	Age (Year)	Height (cm)	Body Mass (kg)	BMI (kg/m^2^)	90% HR_max_
**PH**	26 ± 8	162 ± 4	73 ± 5	27.6 ± 1.9	~171 bpm
**CG**	24 ± 6	163 ± 3	76 ± 11	28.8 ± 4.3	~172 bpm
**SG**	24 ± 6	160 ± 2	77 ± 9	29.9 ± 1.2	~172 bpm

Data are presented in mean ± standard deviation. Abbreviation: PH = placebo and high-intensity interval training (HIIT); CG = combined group (spirulina and HIIT); SG = spirulina group; BMI = body mass index; 90% HR_max_ = 90% of maximum heart rate; bpm = beats per minute.

**Table 2:** Changes in VO_2max_ and immunoglobulins.

Variables	Groups	Pre-Training	Post-Training	CI 95% for Difference		Hedge’s g	% Changes
				Lower	Upper		
**VO_2max_** **(mL·kg^−1^·** **min^−1^)**	*PH*	21.8 ± 3.4	23.4 ± 1.8	−4.21	0.93	0.57	9.21
	*CG*	23.2 ± 2.1	25.6 ± 3.1	−4.85	0.05	0.88	11.03
	*SG*	21.3 ± 1.9	21.9 ± 3.0	−2.91	1.73	0.23	2.58
**IgA** **(mg/lit)**	*PH*	161.7 ± 86.2	149.2 ± 50.8	−53.97	78.97	−0.16	−2.31
	*CG*	173.1 ± 33.7	212.7 ± 67.8 *	−89.90	10.70	0.70	21.23
	*SG*	171.1 ± 47.9	187.5 ± 44.6 *	−59.86	27.06	0.33	11.82
**IgG** **(mg/lit)**	*PH*	1253.5 ± 413.6	1227.1 ± 316.1	−319.46	372.26	−0.06	−3.21
	*CG*	1309.3 ± 285.3	1461.5 ± 240.1	−399.91	95.51	0.55	13.28
	*SG*	1301.0 ± 317.8	1460.4 ± 272.2	−437.38	118.58	0.51	14.17

Abbreviation: VO_2max_ = maximum oxygen consumption; IgA = immunoglobulin A; IgG = immunoglobulin G; HIIT = high-intensity interval training; CI = confidence interval; PH = placebo and HIIT; CG = combined group; SG = spirulina group. * to reflect significance pre- vs. post-test at the level of *p* < 0.05.

**Table 3:** Changes in anthropometric and body composition.

Variables	Groups	Pre-Training	Post-Training	CI 95% for Difference		Hedge’s g	% Changes
				Lower	Upper		
**BMI** **(kg/m^2^)**	*PH*	27.6 ± 1.9	26.3 ± 1.9	−0.48	3.08	−0.65	−4.64
	*CG*	28.8 ± 4.3	27.7 ± 4.6	−3.08	5.26	−0.23	−3.92
	*SG*	29.9 ± 4.0	29.6 ± 4.1	−3.49	4.09	−0.07	−1.06
**WHR** **(cm)**	*PH*	0.80 ± 0.05	0.79 ± 0.06	−0.04	0.06	−0.17	−1.15
	*CG*	0.73 ± 0.03	0.72 ± 0.03	−0.01	0.03	−0.31	−1.79
	*SG*	0.88 ± 0.18	0.80 ± 0.09	−0.05	0.21	−0.53	−6.74
**Body mass** **(kg)**	*PH*	73.3 ± 5.3	70.2 ± 4.0	−1.33	7.45	−0.62	−4.03
	*CG*	76.7 ± 11.1	74.1 ± 11.1	−7.83	13.01	−0.22	−3.40
	*SG*	77.3 ± 8.9	76.1 ± 10.0	−7.64	10.10	−0.12	−1.73
**FFM** **(kg)**	*PH*	19.5 ± 2.1	19.1 ± 2.4	−1.76	2.46	−0.14	−1.89
	*CG*	18.8 ± 0.8	18.3 ± 0.96 *	−0.32	1.36	−0.54	−2.76
	*SG*	19.7 ± 2.3	20.1 ± 2.1	−2.47	1.67	0.17	2.22
**BF (%)**	*PH*	31.7 ± 3.0	30.4 ± 3.0	−1.39	4.09	−0.43	−4.20
	*CG*	32.0 ± 2.4	31.0 ± 3.0	−1.48	3.62	−0.37	−3.43
	*SG*	33.9 ± 3.9	31.3 ± 2.9	−0.64	5.82	−0.71	−7.29

Abbreviation: BMI = body mass index; WHR = waist–hip ratio; FFM = free fat mass; BF% = body fat percentage; HIIT = high-intensity interval training; CI = confidence interval; PH = placebo and HIIT; CG = combined group spirulina and HIIT; SG = spirulina group. * to reflect significance pre- vs. post-test at the level of *p* < 0.05.

## Data Availability

The datasets used and/or analyzed during the current study are available from the corresponding author on reasonable request.
